# A Comparison of Intercostal Nerve Block and Thoracic Epidural Anesthesia in Patients Undergoing Video-Assisted Thoracic Surgery: A Propensity Score-Matched Retrospective Study

**DOI:** 10.7759/cureus.81635

**Published:** 2025-04-02

**Authors:** Kyosuke Takahashi, Mai Yoshimochi, Shigehiko Uchino, Keisuke Kajitani, Kentaro Fukano, Wakako Sato, Yusuke Iizuka, Yuji Otsuka, Koichi Yoshinaga

**Affiliations:** 1 Department of Anesthesiology, Institute of Science Tokyo Hospital, Tokyo, JPN; 2 Department of Anesthesiology, Hamamatsu University School of Medicine, Shizuoka, JPN; 3 Department of Anesthesiology and Critical Care Medicine, Jichi Medical University Saitama Medical Center, Saitama, JPN; 4 Department of Anesthesiology, Mitsui Memorial Hospital, Tokyo, JPN; 5 Department of Anesthesiology and Critical Care Medicine, Jichi Medical University, Shimotsuke, JPN

**Keywords:** epidural anesthesia, peripheral nerve block, regional anesthesia, thoracic surgery, thoracoscopic surgery

## Abstract

Background: Intercostal nerve block (ICNB) plus intravenous (IV) patient-controlled analgesia (PCA) could be an alternative method of perioperative pain management in patients undergoing video-assisted thoracic surgery (VATS). However, the efficacy of this strategy has not been established.

Methods: A retrospective observational study was conducted at an acute care hospital in Japan. Among patients who underwent VATS under general anesthesia from January 1, 2012, to December 31, 2022, we included those who received ICNB or thoracic epidural anesthesia (TEA). The ICNB group had postoperative IV PCA, and the TEA group had postoperative epidural PCA. VATS indicated for pneumothorax or biopsy was excluded. The primary outcome was the maximum pain score measured by the numerical rating scale on postoperative day 1. Secondary outcomes included the times rescue analgesics were used and the use of antiemetics. Propensity score matching was performed to minimize bias from nonrandomized assignment of anesthesia methods.

Results: Among 1,641 patients who met the criteria, 590 underwent ICNB and IV PCA, while 1,051 received TEA. After 1:1 propensity score-matching, 456 were in each group. The median (interquartile range) pain score on postoperative day 1 was higher in the ICNB group than in the TEA group, with values of 5 (4-7) vs. 3 (2-5) (p < 0.0001). Patients in the ICNB group more frequently used rescue analgesics on postoperative day 0, with values of 2 (1-2) vs. 1 (1-2) (p < 0.0001), and had a higher proportion of receiving antiemetics on postoperative day 1 (13.4% vs. 6.1%, p = 0.0004), compared to the patients in the TEA group.

Conclusions: ICNB plus IV PCA was inferior to TEA for postoperative pain management of VATS in the study population. Protocol-based prospective studies are needed to determine the efficacy of this strategy.

## Introduction

Minimally invasive procedures are currently the mainstream of thoracic surgery. A report in 2020 showed that 70.6% of thoracic surgery was performed with the minimally invasive approach, which includes video-assisted thoracic surgery (VATS) and robot-assisted thoracic surgery [1]. Although VATS is generally associated with milder degrees of acute pain than open thoracotomy, the incidence of chronic pain is similar between VATS and thoracotomy [2]. Since higher degrees of postoperative pain are associated with an increased probability of chronic pain [3], perioperative pain management is crucial not only for early recovery but also for long-term quality of life.

Regional anesthesia is a standard for postoperative pain management in VATS. Although thoracic epidural anesthesia (TEA) can provide good pain relief and has been the gold standard in thoracic surgery, peripheral nerve blocks are gaining popularity due to similar analgesic effects and lower risks of adverse events compared to TEA [4]. Meta-analyses showed that paravertebral block and erector spinae block are as effective as TEA, and the incidence of complications was lower [5,6]. Currently, postoperative pain management guidelines recommend that these peripheral nerve blocks are the first line for patients undergoing VATS [7].

Intercostal nerve block (ICNB) could be another option that is safe and effective for minimally invasive thoracic surgery. Surgeons can perform this block intraoperatively under thoracoscopic guidance by identifying the intercostal space [8]. A meta-analysis showed that ICNB was clinically noninferior to TEA, but its opioid-sparing effect was milder [9]. Hence, ICNB may be superior to TEA when it is combined with intravenous (IV) patient-controlled analgesia (PCA). However, the current guidelines state that there are no recommendations on ICNB due to its scarce evidence [7].

This study aims to clarify the efficacy of ICNB plus IV PCA on postoperative pain in patients receiving VATS. We hypothesized that ICNB plus IV PCA may offer comparable analgesic effects to TEA in patients undergoing VATS.

## Materials and methods

Study design

This is a single-center retrospective study conducted in an acute care hospital in Japan. The study was conducted in accordance with the principles of the Declaration of Helsinki for Human Research. The institutional review board of Jichi Medical University Saitama Medical Center approved the study design (certification no. RINS23-092). Informed consent was waived due to the retrospective nature of the study.

Inclusion and exclusion criteria

The eligibility criteria were patients aged 18 or older who underwent VATS from January 1, 2012, to December 31, 2022. Among the eligible patients, we included those who received general anesthesia combined with ICNB or TEA and subsequent postoperative PCA. The exclusion criteria were as follows: pregnancy, multiple regional anesthetics, VATS indicated for pneumothorax or biopsy, conversion to thoracotomy during surgery, ICNB without IV PCA, and TEA without postoperative epidural PCA.

Anesthetic management of VATS in our institution

In our institution, general anesthesia combined with regional anesthesia is performed for patients undergoing VATS. The main options for regional anesthesia were ICNB and TEA. The first line of regional anesthesia was TEA, but it changed to ICNB in 2019 as surgeons requested to shorten the stay in the OR. However, the final choices of anesthesia methods were up to each anesthesiologist’s decision, based on the possibility of converting to thoracotomy, perioperative use of anticoagulants, and patients’ preferences. As for maintenance of general anesthesia, we used propofol or inhalation anesthetics (sevoflurane or desflurane) in combination with remifentanil. All patients were intubated and mechanically ventilated during surgical procedures.

ICNB was performed by surgeons at the end of the thoracoscopic procedure under thoracoscopic guidance. Surgeons determined the levels of intercostal nerves to block according to the thoracoscopy ports used. We used 0.25% levobupivacaine 20-40 mL based on the number of intercostal nerves to block and body weight. Fentanyl up to 5 µg/kg was given during surgery as a loading dose for postoperative IV PCA. The recipe of IV PCA was as follows: drug, fentanyl; background infusion, 20 µg/hour; bolus, 20 µg; and lockout interval, 30 minutes.

Anesthesiologists performed TEA before inducing general anesthesia. The level of catheter insertion was based on the skin incision levels. Intermittent bolus doses of 0.375% ropivacaine, 5-10 mL, were given before and during surgery. Epidural PCA was started at the time as per the anesthesiologists’ decision (drug, 0.125% levobupivacaine with or without fentanyl; background infusion, 4 mL/hour; bolus, 3 mL; lockout interval, 30 minutes).

PCA continued until postoperative day 3 or until the surgeons decided it was no longer needed. Postoperative pain medications included acetaminophen and nonsteroidal anti-inflammatory drugs (NSAIDs, including flurbiprofen, loxoprofen, and celecoxib), and metoclopramide was used as a rescue antiemetic. These medications were administered either at the patient’s request or when deemed necessary by the medical staff.

Data collection and outcomes

We collected data from patients’ electronic medical records at our institution, including anesthesia records. The extracted data included patients’ demographic details, diagnoses, surgical procedures, anesthesia methods, postoperative pain assessment, and postoperative other pain medications and antiemetics. Missing data were not imputed.

We defined the primary outcome as the maximum value of the numerical rating scale (NRS) on postoperative day 1. In the NRS, patients were asked to answer on a scale from 0 to 10. Zero represents “no pain,” while 10 represents “the worst pain imaginable [10].” NRS was assessed by staff nurses in general wards or the intensive care unit, who were not involved in anesthetic management. They assessed NRS at least thrice daily (morning, afternoon, and evening). Secondary outcomes were the maximum values of NRS on postoperative days 0 and 2, the times analgesics were used, the use of acetaminophen and NSAIDs, and the use of antiemetics.

Statistical analysis

Continuous variables were described as means ± standard deviation (SD) or medians with interquartile range (IQR) according to the normality of data. Categorical variables were presented as counts with percentages. The differences in baseline characteristics were expressed as standardized mean differences (SMDs).

We performed a propensity score-matching to compare the outcomes. Potential confounders used for propensity score matching were age, sex, American Society of Anesthesiologists physical status, smoking, diagnosis indicated for surgery, surgical procedure, duration of surgery, and type of anesthesia. The propensity score variables were entered into a multiple regression model, and the likelihood of receiving ICNB was estimated (propensity score). We performed a 1:1 nearest neighbor matching without replacement with a caliper of 0.2 times log (SD of propensity score). The covariate balance was checked before and after matching. If a suitable matched control could not be found, the cases were discarded from further analysis.

The Mann-Whitney U test was used to compare pain scores by NRS and the times pain medications were given. The chi-square test was used for the other outcomes. We considered a p value of less than 0.05 as statistically significant. All statistical analyses were performed using R software version 4.0.3 (R Foundation for Statistical Computing, Vienna, Austria).

## Results

Characteristics of the patients

**Figure 1:**
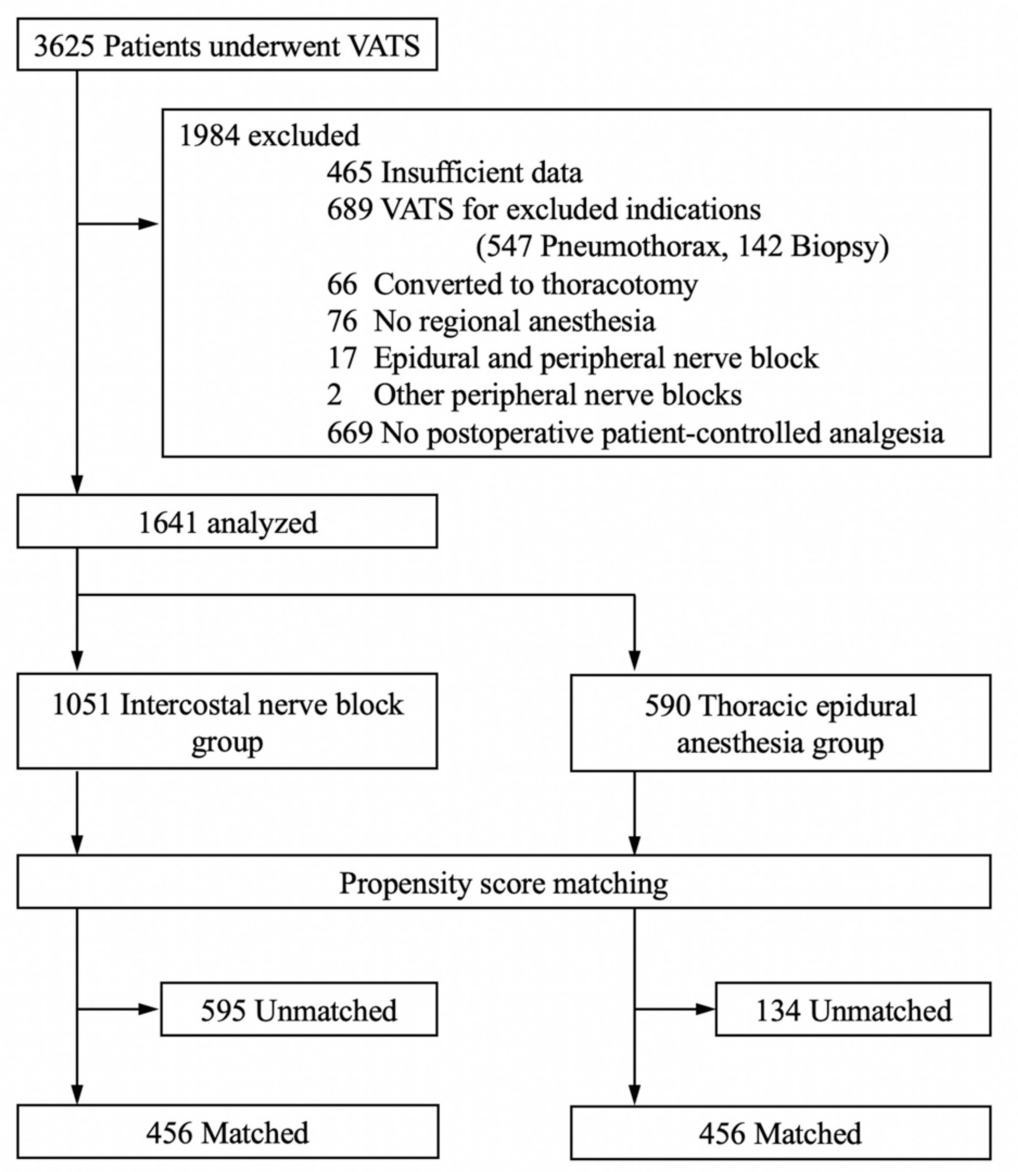
Patient flow diagram VATS: video-assisted thoracic surgery

During the study period, 3,625 patients underwent VATS in our institution. Of these, 1,984 were excluded due to not meeting the criteria or insufficient data (Figure 1). Among 1,641 patients analyzed, 590 (36%) underwent ICNB and IV PCA, while 1,051 (64%) received TEA and epidural PCA. No patient had IV PCA plus epidural PCA postoperatively. The median age of the overall study population was 70 (IQR, 67-76) years, and 972 (59%) were men. Patients diagnosed with lung cancer were 1,314 (80%) among the study population. Smoking until admission was prevalent in 88 (5.4%) patients, and 174 (11%) suffered from chronic pulmonary obstructive disease.

Before propensity score-matching, patients in the ICNB group were older, and the proportions of cancer diagnoses were higher than those in the TEA group. Physical status and surgical procedures also differed between the groups. The patients in the ICNB group were more likely to be classified as higher classes of the American Society of Anesthesiologists Physical Status and had more segmentectomy and wedge resection (Table 1). After 1:1 propensity score matching, 456 were in each group. The differences in characteristics between the groups were reduced to an SMD of <0.1 in all items.

**Table 1:** Characteristics of the study population before and after propensity score-matching ASA PS: American Society of Anesthesiologists physical status; BMI: body mass index; COPD: chronic obstructive pulmonary disease; ICNB: intercostal nerve block; IQR: interquartile range; SMD: standardized mean difference; TEA: thoracic epidural anesthesia; SD: standard deviation; FEV1.0: forced expiratory volume 1.0

Characteristic	Before propensity score-matching			After propensity score-matching		
	ICNB (n = 590)	TEA (n = 1,051)	SMD	ICNB (n = 456)	TEA (n = 456)	SMD
Age (year), median (IQR)	72 (64-77)	69 (62-75)	0.141	72 (64-77)	70 (62-76)	0.075
Male sex (%)	371 (62.9)	601 (57.2)	0.117	280 (61.4)	276 (60.5)	0.018
BMI (kg/m^2^), mean (SD)	23.3 (3.9)	23.3 (3.4)	0.02	23.0 (3.8)	23.4 (3.4)	0.020
Current smoking, n (%)	28 (4.7)	60 (5.7)	0.043	21 (4.6)	20 (4.4)	0.011
COPD, n (%)	62 (11.3)	112 (10.8)	0.016	49 (11.5)	56 (12.5)	0.031
Interstitial pneumonia, n (%)	19 (3.4)	34 (3.3)	0.01	15 (3.5)	20 (4.5)	0.048
Chronic heart failure, n (%)	8 (1.5)	4 (0.4)	0.112	4 (0.9)	1 (0.2)	0.094
FEV1.0 (L), median (IQR)	2.1 (1.8-2.6)	2.2 (1.8-2.7)	0.095	2.1 (1.8-2.6)	2.2 (1.8-2.6)	0.038
ASA PS, n (%)						
1	30 (5.1)	169 (16.1)	0.581	25 (5.5)	26 (5.7)	0.025
2	421 (71.4)	813 (77.4)		360 (78.9)	363 (79.6)	
3	138 (23.4)	69 (6.6)		71 (15.6)	67 (14.7)	
4	1 (0.2)	0 (0)		0 (0)	0 (0)	
Diagnosis, n (%)						
Lung cancer	489 (82.9)	825 (78.5)	0.253	368 (80.7)	358 (78.5)	0.065
Benign lung tumor	23 (3.9)	103 (9.8)		20 (4.4)	22 (4.8)	
Mediastinal tumor	54 (9.2)	85 (8.1)		51 (11.2)	59 (12.9)	
Other tumor	2 (0.3)	9 (0.9)		2 (0.4)	2 (0.4)	
Infectious disease	10 (1.7)	16 (1.5)		9 (2)	8 (1.8)	
Other diagnoses	12 (2)	13 (1.2)		6 (1.3)	7 (1.5)	
Surgical procedure, n (%)						
Lobectomy	309 (52.4)	796 (75.7)	0.659	287 (62.9)	275 (60.3)	0.083
Segmentectomy	97 (16.4)	15 (1.4)		15 (3.3)	15 (3.3)	
Tumor resection	57 (9.7)	88 (8.4)		53 (11.6)	61 (13.4)	
Wedge resection	115 (19.5)	117 (11.1)		92 (20.2)	93 (20.4)	
Bilateral surgery	5 (0.8)	12 (1.1)		2 (0.4)	4 (0.9)	
Others	7 (1.2)	23 (2.2)		7 (1.5)	8 (1.8)	
Sedative during maintenance of anesthesia, n (%)						
Propofol	498 (84.4)	914 (87)	0.073	387 (84.9)	382 (83.8)	0.03
Inhalation anesthetics	92 (15.6)	137 (13)		69 (15.1)	73 (16.2)	
Duration of surgery (minutes), median (IQR)	125 (84.2-163)	135 (98-169)	0.144	126 (84-165)	124 (83-160)	0.055
Duration of anesthesia (minutes), median (IQR)	193.5 (153.2-238)	202 (164-238)	0.123	196 (152-238.2)	191 (148.8-232)	0.066
Level of epidural catheter placement, n (%)						
Th4/Th5	0 (-)	53 (5.2)	-	0 (-)	20 (4.5)	-
Th5/Th6	0 (-)	378 (37.4)		0 (-)	167 (38)	
Th6/Th7	0 (-)	398 (39.3)		0 (-)	181 (41.1)	
Th7/Th8	0 (-)	170 (16.8)		0 (-)	68 (15.5)	
Th8/Th9	0 (-)	10 (1)		0 (-)	3 (0.7)	
Th9/Th10	0 (-)	1 (0.1)		0 (-)	1 (0.2)	
Th10/Th11	0 (-)	1 (0.1)		0 (-)	0 (0)	
Th12/L1	0 (-)	1 (0.1)		0 (-)	0 (0)	

Postoperative pain

**Figure 2:**
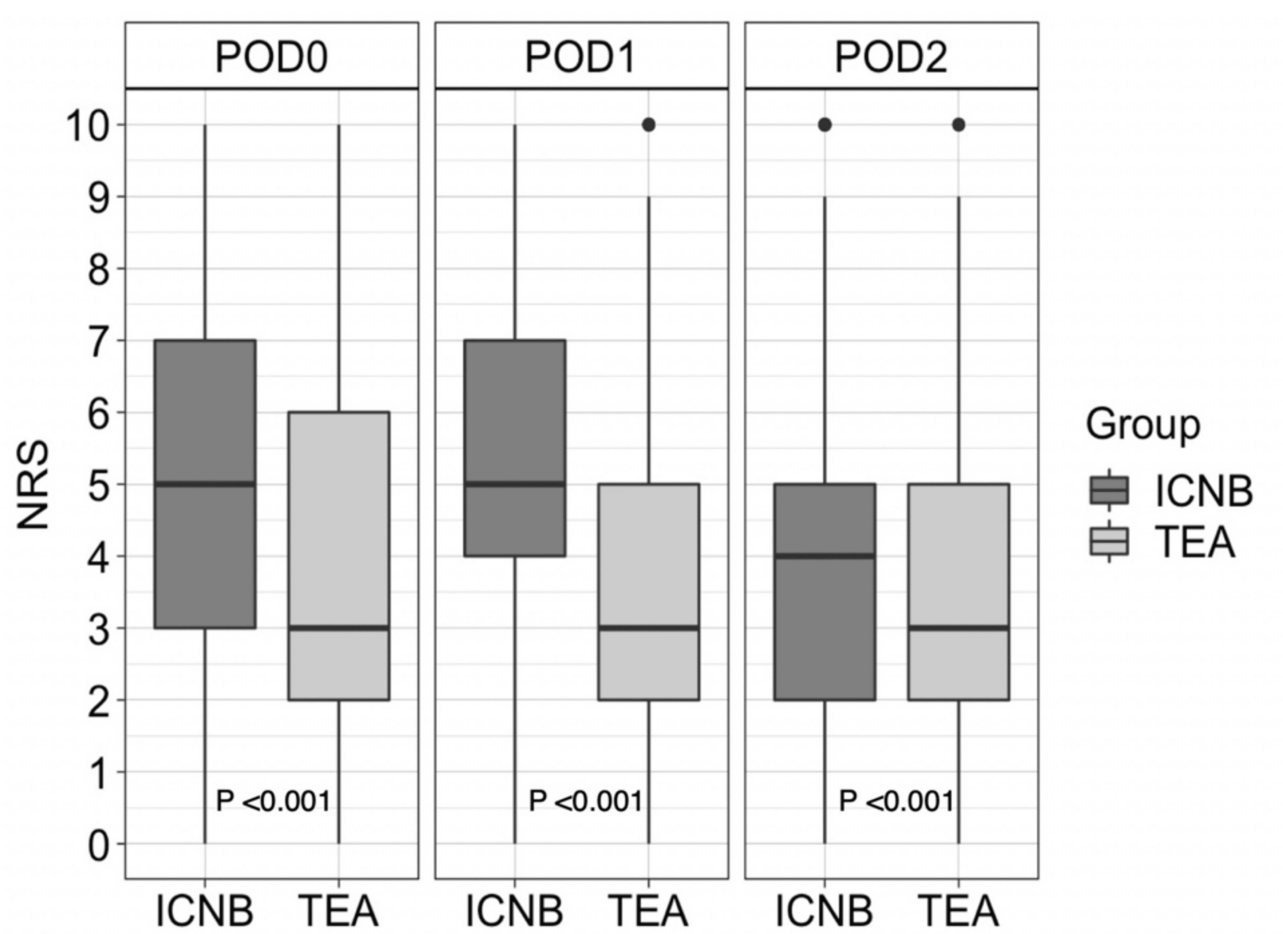
Maximum values of the NRS on postoperative days 0-2 The Mann-Whitney U test was used to compare pain scores by NRS ICNB: intercostal nerve block; NRS: numerical rating scale; POD: postoperative day; TEA: thoracic epidural anesthesia

Figure 2 shows the maximum values of the NRS on postoperative days 0-2 in the propensity-matched groups. Maximus values were significantly higher in the ICNB group than in the TEA group. The median (IQR) values were 5 (3-7) vs. 3 (2-6) on postoperative day 0 (p < 0.0001), 5 (4-7) vs. 3 (2-5) on postoperative day 1 (p <0.0001), and 4 (2-5) vs. 3 (2-5) on postoperative day 2 (p < 0.0001), respectively.

Use of postoperative medication

Table 2 describes the use of medications in the postoperative period. Overall, the number of patients who received any analgesics on postoperative days 0-2 was 752 (83%), 629 (69%), and 157 (17%), respectively, which did not significantly differ between the groups. The median (IQR) times of analgesics used on postoperative day 0 were 2 (1-2) in the ICNB group and 1 (1-2) in the TEA group, which showed a significant difference (p < 0.0001). The difference was still significant on postoperative day 1 (p = 0.007) but not on postoperative day 2 (p = 0.117).

**Table 2:** Medications used on postoperative days 0-2 The Mann-Whitney U test was used to compare the times pain medications were given. The chi-square test was used for the other outcomes ICNB: intercostal nerve block; NSAIDs: nonsteroidal anti-inflammatory drugs; POD: postoperative day; TEA: thoracic epidural anesthesia; IQR: interquartile range

Outcome	Overall (n = 912)	ICNB (n = 456)	TEA (n = 456)	p value	Statistic value
Acetaminophen, n (%)					
POD0	483 (53)	319 (70)	164 (36)	<0.0001	104.38
POD1	351 (38.5)	219 (48)	132 (28.9)	<0.0001	34.255
POD2	113 (12.4)	73 (16)	40 (8.8)	0.001	10.344
NSAIDs, n (%)					
POD0	644 (70.6)	322 (70.6)	322 (70.6)	>0.9999	0
POD1	360 (39.5)	164 (36)	196 (43)	0.036	4.4104
POD2	54 (5.9)	23 (5)	31 (6.8)	0.326	0.9645
Any analgesics, n (%)					
POD0	752 (82.5)	378 (82.9)	374 (82)	0.794	0.0682
POD1	629 (69)	326 (71.5)	303 (66.4)	0.115	2.4797
POD2	157 (17.2)	87 (19.1)	70 (15.4)	0.161	1.9696
Times analgesics given, median (IQR)					
POD0	1 (1-2)	2 (1-2)	1 (1-2)	<0.0001	76,342
POD1	1 (0-1)	1 (0-1)	1 (0-1)	0.007	94,396
POD2	0 (0-0)	0 (0-0)	0 (0-0)	0.117	99,879
Antiemetics, n (%)					
POD0	68 (7.5)	44 (9.6)	24 (5.3)	0.017	5.737
POD1	89 (9.8)	61 (13.4)	28 (6.1)	0.0004	12.75
POD2	17 (1.9)	9 (2)	8 (1.8)	>0.9999	0

As for the type of postoperative medications, acetaminophen was more frequently used in the ICNB group than in the TEA group from postoperative days 0-2 (p < 0.0001). NSAIDs were used significantly in the ICNB group on postoperative day 1 (p = 0.0036), but the difference was not significant on the other days. Those who received antiemetics were more frequent in the ICNB group than in the TEA group on postoperative day 0, i.e., 44 (9.6%) vs. 24 (5.3%), p = 0.017, and day 1, i.e., 61 (13.4%) vs. 28 (6.1%), p = 0.0004.

## Discussion

This propensity score-matched cohort study compared the postoperative outcomes between ICNB plus IV PCA and TEA in patients with VATS. We found that the patients who received ICNB plus IV PCA had higher postoperative pain scores than those who received TEA. The requirement for pain medications and antiemetics was also higher in the ICNB plus IV PCA group.

In previous studies, the effectiveness of ICNB has been compared with other regional anesthesia techniques. A retrospective study, which included 108 patients, compared TEA and thoracoscopy-guided ICNB with liposomal bupivacaine and found no difference in postoperative pain scores [11]. On the other hand, ICNB resulted in higher pain scores, particularly for dynamic pain, compared to TEA in randomized controlled trials of thoracotomy [12,13]. Our findings also indicated that ICNB was inferior to TEA in postoperative pain relief of thoracoscopic surgery. Regarding the comparisons among peripheral nerve blocks, a randomized controlled trial that compared ICNB and paravertebral block in VATS revealed no significant difference between the two blocks for postoperative acute and chronic pain [14]. In another study that compared three peripheral nerve blocks in thoracoscopic pulmonary surgery, ICNB failed to show superiority to paravertebral block, but the effects were similar between ICNB and erector spinae plane block [15]. Considering the findings of our study and the previous reports, the analgesic effects of ICNB may be similar to other peripheral nerve blocks, but not as much as TEA.

Chronic pain is a common issue after thoracic surgery, which affects the postoperative course and patients’ quality of life. A large systematic review and meta-analysis showed the incidence of chronic pain at six months was as high as 47% [16]. Since intense acute pain is associated with chronic pain [3], management in the acute phase is crucial. In this context, TEA is an established method for the prevention of chronic pain. A Cochrane review including 23 studies demonstrated that TEA reduced the risk of developing chronic pain after thoracotomy in one patient out of every four patients treated [17]. ICNB was also reported to reduce chronic postsurgical pain after lung surgery [18]. However, a randomized controlled trial that directly compared ICNB and TEA in lung cancer resection revealed that those who received ICNB had a higher incidence of chronic postthoracotomy pain syndrome [19]. Taking these findings into account, the modest analgesic effects of ICNB may contribute to a milder reduction in long-term pain after thoracic surgery.

ICNB is easy to perform under thoracoscopic guidance. Surgeons can directly confirm the expansion of the visceral pleura while injecting a local anesthetic, which makes this block safe and accurate [8]. The complications of ICNB include bleeding, local anesthetic toxicity, and nerve injury, but the incidence of overall adverse events is possibly lower than that of TEA. A retrospective study including 116 patients with rib fractures showed that 26% of patients who received TEA experienced complications, while those who underwent ICNB had no complications [20]. Urinary retention and hypotension are common side effects of epidural block [21,22], and these could affect the postoperative course of pulmonary surgery. Several studies reported longer hospital stays in patients who received TEA than those who had ICNB [11,23]. Additionally, serious complications such as epidural hematoma and permanent nerve injury could occur. Due to the possible complications and side effects, routine use of TEA is currently not recommended [7]. Given that the impact of adverse events could outweigh its merits in pain relief, the indications of TEA in VATS may be limited to cases that have a high probability of conversion to thoracotomy or a high risk for acute and chronic pain.

Multimodal analgesia is the standard strategy for postoperative pain management. In addition to regional anesthesia, systemic analgesia, including acetaminophen and NSAIDs, is a component of pain management in VATS. A study including 99 patients undergoing VATS investigated the effects of acetaminophen combined with TEA. The patients who received scheduled IV acetaminophen had lower pain scores compared to those who did not have acetaminophen [24]. Several randomized controlled trials also showed that combinations of a peripheral nerve block and an IV NSAID resulted in low postoperative pain scores in thoracoscopic surgery [25,26]. Although these drugs were used as additional medications in our study, the scheduled use of acetaminophen and NSAIDs could be an option to enhance analgesic effects.

Postoperative nausea and vomiting (PONV) is a major concern that could affect postoperative recovery. The incidence of PONV in lung surgery is reported as approximately 20%-30% [27,28], which can hinder early mobilization. A systematic review and meta-analysis found no significant difference in the incidence of PONV when comparing peripheral nerve blocks to epidural analgesia [29]. However, the use of fentanyl is identified as a risk factor for PONV, and increased doses are related to the higher incidence [28]. Similarly, our study found that antiemetics were more frequently used postoperatively in the ICNB plus IV PCA group than in the TEA group. This finding indicated that the use of fentanyl IV PCA could increase the incidence of PONV. Although combinations of regional anesthesia and opioids are common approaches for postoperative pain management, the doses of opioids may need to be adjusted according to the patient's profile. Considering that the opioid-sparing effect of ICNB is milder than TEA, multimodal regimens, including nonopioid analgesics, should be reasonable when ICNB is performed for thoracic surgery.

This study has several strengths. To the best of our knowledge, the sample size is the largest among studies that compared regional anesthesia techniques in VATS. As this study was conducted in a high-volume center of thoracic surgery, we believe that surgical procedures were consistent, which could contribute to the fair comparison of the anesthesia methods in the real-world setting. However, our study is limited by its retrospective nature. Unknown confounding factors might have affected the results, although we performed propensity score matching to mitigate this issue. Another problem was that we were unable to collect data for complications of regional anesthesia, such as postoperative hypotension, nerve injury, and local anesthetic toxicity. While the analgesic effects of TEA were superior to ICNB, the complications may have been more frequent in TEA than in ICNB.

## Conclusions

ICNB plus IV PCA was inferior to TEA for postoperative pain scores of VATS in the study population. The requirement for pain medications and antiemetics was also higher in the ICNB plus IV PCA group. These findings may indicate that some patient populations could be more suitable to receive TEA rather than ICNB for thoracoscopic surgery. Strategy for postoperative pain management may need to be individualized based on patient profile, surgical procedures, and possible adverse events of regional anesthesia. Further protocol-based prospective studies are needed to determine the efficacy of these anesthetic techniques.
